# Any neuron can perform linearly non-separable computations

**DOI:** 10.12688/f1000research.53961.2

**Published:** 2021-09-16

**Authors:** Romain D. Cazé

**Affiliations:** 1 CNRS IEMN UMR 8520, Villeneuve d'ascq, Haut de France, 59650, France

**Keywords:** Dendrites, computation, linearly non-separable, neuroscience

## Abstract

Multiple studies have shown how dendrites enable some neurons to perform linearly non-separable computations. These works focus on cells with an extended dendritic arbor where voltage can vary independently, turning dendritic branches into local non-linear subunits. However, these studies leave a large fraction of the nervous system unexplored. Many neurons, e.g. granule cells, have modest dendritic trees and are electrically compact. It is impossible to decompose them into multiple independent subunits. Here, we upgraded the integrate and fire neuron to account for saturating dendrites. This artificial neuron has a unique membrane voltage and can be seen as a single layer. We present a class of linearly non-separable computations and how our neuron can perform them. We thus demonstrate that even a single layer neuron with dendrites has more computational capacity than without. Because any neuron has one or more layer, and all dendrites do saturate, we show that any dendrited neuron can implement linearly non-separable computations.

## Introduction

We show here how dendrites can extend the computational capacity of all neurons, even the tiniest. We already knew that dendrites might extend the computational capacity of some pyramidal neurons. Their extended dendrites capable of dendritic spikes changed the way we saw them (see ^2^ for one of the first articles presenting this idea). More recently a study suggested that we should model these neurons as a two layer neural networks. ^6^ This theoretical model was further consolidated by experiments showing that we can see a pyramidal neuron as a collection of non-linear subunits. ^7^ Certain non-linearities can even allow a dendrite to implement the exclusive or (XOR). ^9^ Moreover, a similar kind of non-monotonic non-linearity was found in human pyramidal neurons. ^4^ But what about other neurons with modest dendrites incapable of spiking?

Pyramidal neurons only represent a fraction of all neurons. For instance, the dendrites of cerebellar stellate cells cannot emit spikes, but they do saturate ^1^ and they can be decomposed into multiple independent subunits - with independent membrane voltages - turning them into two-stage units like the pyramidal neuron. ^8^ Previously we have shown that passive dendrites are sufficient to enable a neuron to perform linearly non-separable computations, for instance, the feature binding problem. ^3^ We focus here on cells with a modest and passive dendritic tree. These cells form a single layer unit. In the present study, we demonstrate that these neurons can still implement a linearly non-separable computation. We use them as the simplest common denominator, as even spiking dendrites do saturate, and a 2 layer network can perform all the computation of a single layer architecture and more.

## Methods

### An integrate and fire neuron with dendrites (the DIF)

We started from a leaky integrate and fire (LIF). This model has a membrane *V* modelled by the following equation:

$$\tau \frac{dv}{dt}=(v_{E}-\;v(t))+RI_{s}(t)\;$$ (1)

With *τ* = 20 ms the neuron time constant, *v*( *t*) the membrane voltage at time *t* and *v*_*E*_ = −62 mV which sets the resting membrane voltage. *R* = 20 MΩ is the value of the resistance and *I*_*s*_( *t*) models the time varying synaptic inputs conductance.

$$I_{s}(t)=\underset{i}{\sum }g_{d^{i}}(t)(E_{s}-v(t))$$ (2)

**Figure 1.**
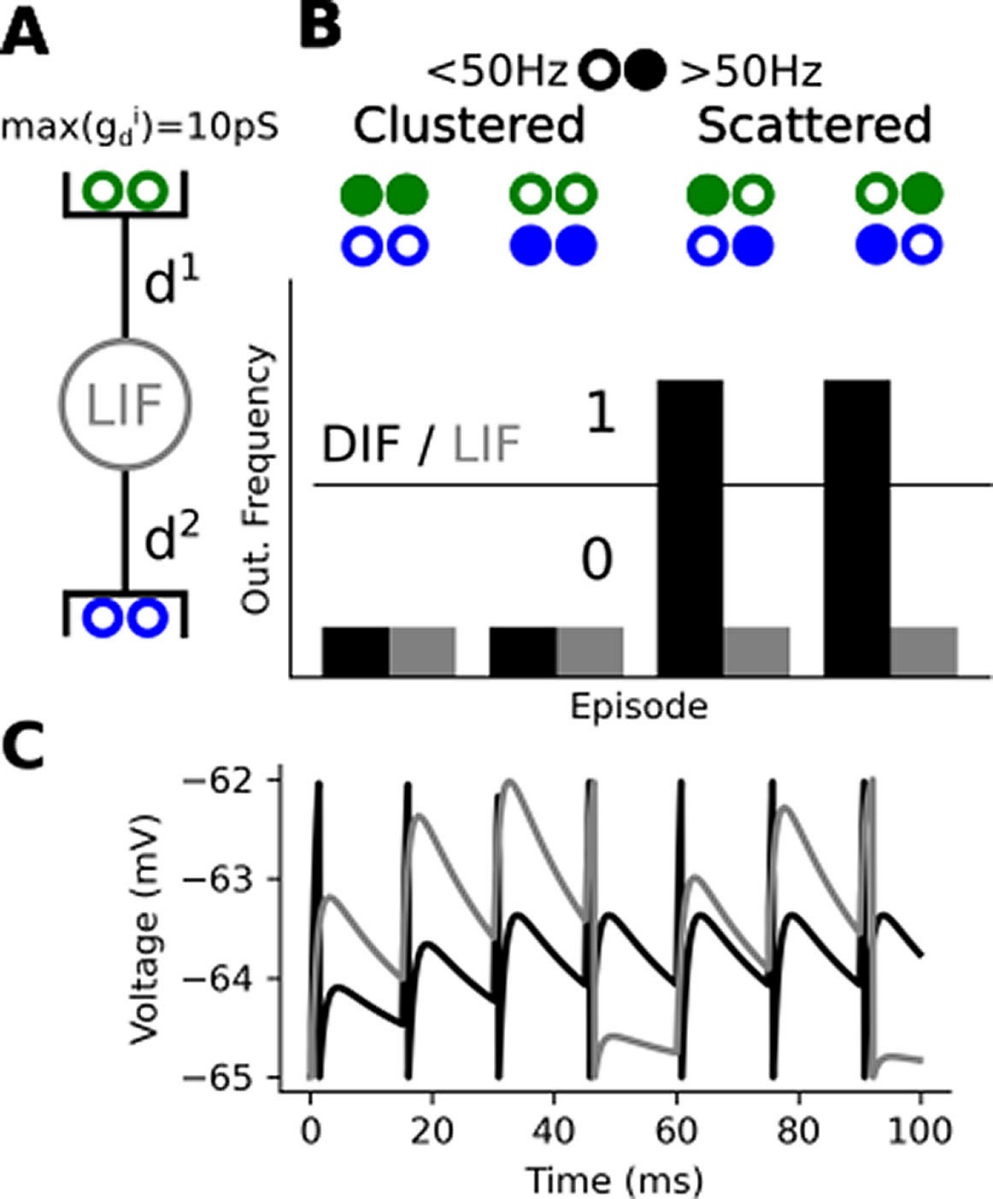
A dendrited integrate and fire implementing a linearly non-separable computation. (A) A leaky integrate and fire (LIF) with two dendrites making it a dendrited integrate and fire (DIF), each half of the 4 synaptic inputs targets a distinct dendrite where *g* locally saturates at 10 *pS* (B) Four stimulation episodes, filled circles stand for a >50 Hz input spike train while empty circles stand for >50 Hz input spike train. Below, we plotted the response of the DIF (black) and a LIF (grey) during the episode. We purposely removed the ticks label as the frequencies depend on the parameter of the model and input regularity. The parameters of the model can vary largely without affecting the observation. (C) Some voltage response during the 3rd episode. In the clustered case (grey), the neuron reach spike threshold three times whereas it reaches spike thresold seven times in the scattered case (black). ^5^

This current depends on the difference between *v*( *t*) the neuron voltage, equal everywhere, and *E*_*s*_ the synaptic reversal potential (0 mV) while $g_{d^{i}}$ is the synaptic conductance in dendrite *i.* Each $g_{d^{i}}$ is bounded between 0 and 10 *pS.* Each $g_{d^{i}}$ jumps up instantaneously to its maximal value for each incoming input spike and decays exponentially with time constant *τ*_*s*_ = 1 ms. In a LIF all synaptic inputs are gathered into a single umbrella and *i* = 1. In the present study, we introduce the Dendrited Integrate and Fire (DIF) which includes at least two dendrites ( *i* = 2). We cluster synaptic inputs into two groups, each targeting a dendrite (one green and one blue, see Figure 1). We used the Brian software version 2 to carry out our simulations, the code is freely available on the git repository attached with this report. ^10^

### Boolean algebra refresher

First, let’s present Boolean functions:

**Definition 1.** *A Boolean function of n variables is a function on* {0,1} ^*n*^ *into* {0,1}, *where n is a positive integer.*

Importantly, we commonly assume that neurons can only implement linearly separable computations:

**Definition 2.** *f is a linearly separable computation of *n* variables if and only if there exists at least one vector* *w* ∈ $R^{n}$ *and a threshold* Θ ∈ R *such that:*

$$f(X)=\left\{\begin{array}{ll} 1\; & \text{if }w\cdot X\geq \Theta \\ 0\; & \text{otherwise} \end{array}\right.$$

*where* *X* ∈{0,1} ^*n*^ *is the vector notation for the Boolean input variables.*

## Results

### The compact feature binding problem (cFBP)

In this section, we demonstrate a class of compact linearly inseparable (non-separable) computations that we are going to study. These computations are compact because they have four input/output lines.

We entirely specify an example in Table 1. This computation that we call the compact feature binding problem (cFBP) is linearly inseparable.

**Table 1.** The truth table of a non-linearly separable computation.

Inputs	Output
0011	0
1100	0
0101	1
1010	1

**Proposition 1.** *The cFBP is linearly inseparable (non-separable)*

*Proof.* The output must be 0 for two disjoint couples (1,2) and (3,4) of active inputs. It means that *w*_1_ + *w*_2_ ≤ Θ, and *w*_3_ + *w*_4_ ≤ Θ, and we can add these two inequalities to obtain *w*_1_ + *w*_2_ + *w*_3_ + *w*_4_ ≤ 2Θ. However, the output must be 1 for two other couples made of the same active inputs (1,3) and (2,4). It means that *w*_1_ + *w*_3_ > Θ, and *w*_2_ + *w*_4_ > Θ, and we can add these two inequalities to obtain *w*_1_ + *w*_2_ + *w*_3_ + *w*_4_ > 2Θ. This yield a contradiction proving that no weights set exists solving this set of inequalities.

The cFBP is simple in two ways:

• Four input/output relations define this computation - the same number as the famous XOR (exclusive or).
• Contrary to the XOR it can be implemented with excitatory inputs and a monotone transfer function. ^3^

We can extend the cFBP by increasing the number of inputs. In this case we deal with tuples instead of couples. As such, the cFBP corresponds to an entire family of linearly inseparable computations, and a dendrited neuron can implement them using the strategy that we will present in the next section.

A LIF with its linear integration cannot implement such a computation. While a neuron with two saturating dendrites can easily implement it. We already proved how a ball-and-stick biophysical model can implement this computation in a previous study. ^3^

### Implementing the cFBP in a dendrited integrate and fire

We use two independently saturating conductances to implement the cFBP in a minimal extension of the LIF that we called the dendrited integrated and fire (DIF). The DIF has a single membrane voltage to account for its compactness so we might wonder how local saturation can arise in such a morphology. Saturation has two possible origins: (1) a reduction in driving force can cause saturation as in, ^1^ but (2) it can also be due to the intrinsic limitations in conductance per unit of surface. This latter possibility makes saturation possible in an electrically compact neuron. Even in a neuron with a small dendritic tree, the conductance is going to reach an upper bound per unit of surface and the only possibility to increase excitation consists in stimulating a larger area. We are going to employ this local bounding of the conductance to implement the cFBP in a DIF.

To do that, we only need two dendrites as shown in Figure 1A. We can interpret the 0s and 1s in the truth table in at least two ways: (1) either the pre- or post-synaptic neurons activates (2) or they reach a given spike frequency. In the following section, we will use the latter interpretation. Consequently, we consider a pre-synaptic input active when it fires above 50 Hz regular spike-train and inactive if it fires below 50 Hz (this value is arbitrary and can largely vary to match a neuron working range). We stimulate our model in four distinct episodes to reproduce the truth table from the previous section. You can observe on Figure 1 that locally bounding *g* enables implement the of cFBP. When *g* has no bound, the membrane voltage always reaches the spiking threshold at the same speed (LIF case). When we locally bound conductances the membrane voltage takes more time (45 ms see Figure 1C) to reach threshold in the clustered case (total *g* = 10 *pS*) than in the scattered case (total *g* = 20 *pS*). All in all, a DIF will respond differently for the clustered and scattered case while a LIF won’t. This enables a DIF to implement the cFBP while a LIF can’t.

## Discussion/conclusion

In this brief report, we introduced a small extension to the leaky integrate and fire neuron: a dendrited integrate and fire neuron which can implement linearly non-separable computations. This single layer model applied to cerebellar granule cells predicts that they can implement a linearly non-separable computation. These neurons have on average four dendrites, but we have shown here that two suffice. The DIF’s multiple distinctly bounded *g* underlie this ability. For example, we need a local saturation of $g_{d^{i}}$ to implement the cFBP.

Importantly, a reduction in driving force does not generate sublinear summation in a DIF. The implementation of a linearly inseparable computation would have been impossible in a single compartment neuron because of interaction via the unique membrane potential. The usage of locally bounded *g* is crucial to make our prediction possible.

The experiment demonstrating this prediction seems straightforward. One would need to stimulate four distinct groups of mossy fibres following our different scenarios. We could then record how a group of granule cell respond using optogenetics reporting (i.e. calcium imaging). We predict that a significant part of granule cells might implement the cFBP. This prediction could reveal the true potential of single neurons. The next step consists of looking at the network level as already done with spiking dendrites. ^5^

## Data availability

No data are associated with this article.

## Software availability

• Source code available from: https://github.com/rcaze/21_03Ca/tree/1.
• Archived source code: https://doi.org/10.5281/zenodo.5355354. ^10^
• License: MIT license.
