# All neurons can perform linearly non-separable computations

**DOI:** 10.12688/f1000research.53961.3

**Published:** 2022-06-08

**Authors:** Romain D. Cazé

**Affiliations:** 1 CNRS IEMN UMR 8520, Villeneuve d'ascq, Haut de France, 59650, France

**Keywords:** Dendrites, computation, linearly non-separable, neuroscience

## Abstract

Multiple studies have shown how dendrites enable some neurons to perform linearly non-separable computations. These works focus on cells with an extended dendritic arbor where voltage can vary independently, turning dendritic branches into local non-linear subunits. However, these studies leave a large fraction of the nervous system unexplored. Many neurons, e.g. granule cells, have modest dendritic trees and are electrically compact. It is impossible to decompose them into multiple independent subunits. Here, we upgraded the integrate and fire neuron to account for saturation due to interacting synapses. This artificial neuron has a unique membrane voltage and can be seen as a single layer. We present a class of linearly non-separable computations and how our neuron can perform them. We thus demonstrate that even a single layer neuron with interacting synapses has more computational capacity than without. Because all neurons have one or more layer, we show that all neurons can potentially implement linearly non-separable computations.

## Introduction

We show here how interaction between synapses can extend the computational capacity of all neurons, even the tiniest. We already knew that dendrites might extend the computational capacity of some pyramidal neurons. Their extended dendrites capable of dendritic spikes changed the way we saw them (see Ref. 1 for one of the first articles presenting this idea). More recently a study suggested that we should model pyramidal neurons as a two layer neural networks. ^2^ This theoretical model was further consolidated by experiments showing that we can see a pyramidal neuron as a collection of non-linear subunits. ^3^ Certain non-linearities can even allow a dendrite to implement the exclusive or (XOR). ^4^ Moreover, a similar kind of non-monotonic non-linearity was found in human pyramidal neurons. ^5^ But what about other neurons with modest dendrites incapable of spiking?

Pyramidal neurons only represent a fraction of all neurons. For instance, the dendrites of cerebellar stellate cells cannot emit spikes, but they do saturate ^6^ and they can be decomposed into multiple independent subunits - with independent membrane voltages - turning them into two-stage units like the pyramidal neuron. ^7^ Previously we have shown that passive dendrites are sufficient to enable a neuron to perform linearly non-separable computations, for instance, the feature binding problem. ^8^ Here, we go one step further and focus on cells with a modest and passive dendritic tree. We use the fact that even in this case spatially nearby synapses can interact due to glutamate spillover (for review see Ref. 9). We show that these cells despite having a single voltage can compute linearly non-separable functions. In the present study. We use these neurons as the smallest common denominator, and we thus conclude that all neurons can perform linearly non-separable functions.

## Methods

### An integrate and fire neuron with interacting synapses (the SIF)

We started from a leaky integrate and fire (LIF). This model has a membrane *V* modelled by the following equation:

$$\tau \frac{dv}{dt}=-(v(t)-v_{E})+RI_{s}(t)\;$$ (1)

With *τ* = 20 ms the neuron time constant, *v*( *t*) the membrane voltage at time *t* and *v*_*E*_ = −65 mV which sets the resting membrane voltage. *R* = 20 MΩ is the value of the resistance and *I*_*s*_( *t*) models the time varying synaptic inputs current. Each time the voltage reaches *V*_*t*_ = −62 mV a spike is triggered and the voltage is resetted to −65 mV. We used the following equation to account for the synaptic inputs.

$$I_{s}(t)=(g_{d^{1}}(t)+g_{d^{2}})(E_{s}-v(t))$$ (2)

**Figure 1.**
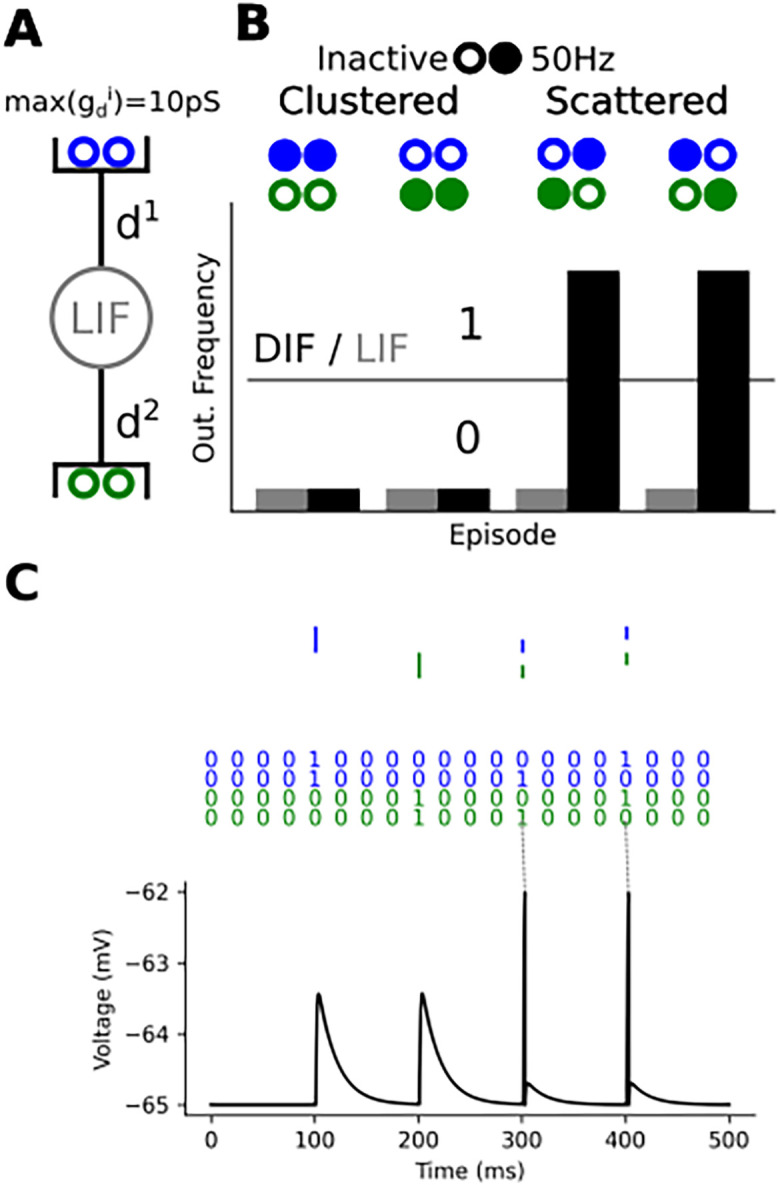
A dendrited integrate and fire implementing a linearly non-separable computation. (A) A leaky integrate and fire (LIF) with two saturating points, each half of the 4 synaptic inputs targets a distinct point where *g* locally saturates at 100 pS (B) Four stimulation episodes, filled circles stand for 50 Hz poisson spike trains while empty circles stand for no input spike train. Below, we plotted the response of the LIF (grey) and of the SIF (black) during an episode. We purposely removed the ticks label as the frequencies depend on the parameter of the model and input correlation. The parameters of the model can vary largely without affecting the observation. (C) Somatic voltage response of the SIF when filled circle means spike and empty circle means no-spike. One can observe that the SIF reproduces the truth table also in this case.

The synaptic current depends on the difference between *v*( *t*) the neuron voltage, equal everywhere, and *E*_*s*_ the synaptic reversal potential (0 mV). In the present work, we have four input sources and contrary to what is done usually we have only two conductances *g*_1_ and *g*_2_ which collapse conductance from input 1,2 and 3,4 respectively. This account for the interaction between the input sources and do not consider them fully independent as it is usually the case. Each *g*_*i*_ is bounded between 0 and 100 pS. Each *g*_*i*_ jumps up instantaneously to its maximal value for each incoming input spike and decays exponentially with time constant *τ*_*s*_ = 1 ms. In a LIF all synaptic inputs are gathered into a single umbrella and *i* = 1. In the present work we cluster synaptic inputs into 2 groups (one green and one blue, see Figure 1). We used the Brian software version 2 to carry out our simulations, the code is freely available on the git repository attached with this report.

### Boolean algebra refresher

First, let’s present Boolean functions:

**Definition 1.** *A Boolean function of n variables is a function on* {0, 1} ^*n*^ *into* {0, 1}, *where n is a positive integer.*

Importantly, we commonly assume that neurons can only implement linearly separable computations:

**Definition 2.** *f is a linearly separable computation of n variables if and only if there exists at least one vector w* ∈ $R^{n}$ *and a threshold* Θ ∈ R *such that:*

$$f(X)=\left\{\begin{array}{ll} 1\; & \text{if }w\cdot X\geq \Theta \\ 0\; & \text{otherwise} \end{array}\right.$$

*where* *X* ∈ {0, 1} ^*n*^ *is the vector notation for the Boolean input variables.*

## Results

### The compact feature binding problem (cFBP)

In this section, we demonstrate a class of compact linearly inseparable (non-separable) computations that we are going to study. These computations are compact because they only need to be defined on have four input lines. Changing the other input lines would not affect our result.

We entirely specify an example in Table 1. This computation that we call the compact feature binding problem (cFBP) is linearly inseparable.

**Table 1.** The truth table of a linearly inseparable computation.

Inputs	Output
0011	0
1100	0
0101	1
1010	1

**Proposition 1.** *The cFBP is linearly inseparable (non-separable)*

*Proof.* The output must be 0 for two disjoint couples (1,2) and (3,4) of active inputs. It means that *w*_1_ + *w*_2_ ≤ Θ, and *w*_3_ + *w*_4_ ≤ Θ, and we can add these two inequalities to obtain *w*_1_ + *w*_2_ + *w*_3_ + *w*_4_ ≤ 2Θ. However, the output must be 1 for two other couples made of the same active inputs (1,3) and (2,4). It means that *w*_1_ + *w*_3_ > Θ, and *w*_2_ + *w*_4_ > Θ, and we can add these two inequalities to obtain *w*_1_ + *w*_2_ + *w*_3_ + *w*_4_ > 2Θ. This yield a contradiction proving that no weights set exists solving this set of inequalities.

The cFBP is compact beauce it specifies only four lines of a function. A complete definition would include 16 distinct input/output relationship. This incomplete definition of the function leaving all the remaining input/output relation is the minimal. This computation is as complex as the famous exclusive OR (XOR). Note here that our SIF can also implement the XOR using a parameter set explained here [?]. However, contrary to the XOR it can be implemented with excitatory inputs and a monotone transfer function. ^8^

We can extend the cFBP by increasing the number of inputs. In this case we deal with tuples instead of couples. As such, the cFBP corresponds to an entire family of linearly inseparable computations, and a SIF can implement them using the strategy that we will present in the next section.

A LIF with its linear integration cannot implement such a computation. While a neuron with two groups of saturating synapses can easily implement it. We already proved how a ball-and-stick biophysical model can implement this computation in a previous study. ^8^

### Implementing the cFBP in a saturating integrate and fire (SIF)

We use two independently saturating conductances to implement the cFBP in a minimal extension of the LIF. The SIF has a single membrane voltage to account for its compactness so we might wonder how local saturation can arise in such a morphology. Saturation has two possible origins: (1) a reduction in driving force can cause saturation as in Ref. 6, but (2) it can also be due to the intrinsic limitations in conductance per unit of surface. This latter possibility makes saturation possible in an electrically compact neuron. In every cases the conductance is going to reach an upper bound per unit of surface and the only possibility to increase excitation consists in stimulating a larger area. We are going to employ this local bounding of the conductance to implement the cFBP in a SIF.

To do that, we only need two saturating points as shown in Figure 1A. We can interpret the 0 s and 1 s in the truth table in at least two ways: (1) either the pre- or post-synaptic neurons activates (2) or they reach a given spike frequency. In the following section, we will use the two interpretations. We first consider a pre-synaptic input active when it fires a 50 Hz poisson spike-train and inactive if it does not fire (this value is arbitrary and can largely vary to match a neuron working range). We stimulate our model in four distinct episodes to reproduce the truth table from the previous section. You can observe on Figure 1 two interpretation of the truth table: either a rate based or a spike based interpretation. In both cases we ca observe that locally bounding g enables to implement the cFBP. When *g* has no bound, the membrane voltage always reaches the spiking threshold at the same speed (LIF case). When we locally bound conductances the total input current is higher if inputs target two points rather than one (total *g* = 100 pS Vs *g* = 200 pS). All in all a SIF will respond differently for the clustered and scattered case while a LIF won’t. This enables a SIF to implement the cFBP while a LIF can’t.

## Discussion/Conclusion

In this brief report, we introduced a small extension to the leaky integrate and fire neuron: a saturating integrate and fire neuron which can implement linearly non-separable computations. Moreover, we have shown here that two saturating points suffice. The SIF’s multiple distinctly bounded *g* underlie this ability.

Importantly, a reduction in driving force is not the main actor triggering sublinear summation in a SIF. The threshold value guarantees *V*_*t*_ = −62 mV that we are always far from the equilibrium voltage of the synapse *E*_*v*_ = 0 mV. Furthermore a granule cell has a single membrane voltage through wich saturating groups of synapses would interactundermining their parallel processing. This would also be the case if the conductance were voltage-gated. The implementation of a linearly inseparable computation would have been impossible in a single compartment neuron because of interaction via the unique membrane potential. The usage of locally bounded *g* is crucial to make our prediction possible.

The experiment demonstrating this prediction seems straightforward. One would need to stimulate four independent groups of mossy fibres following our different scenarios. We could then record how a group of granule cell respond. This can be done using optogenetics reporting (i.e. calcium imaging). We predict that a significant part of Granule cells might implement the cFBP. This prediction could reveal the true potential of single neurons. The next step consists of looking at the network level as already done with spiking dendrites. ^10^ The origin of the sublinearity might not be certain, but it would be certain that these neurons implement a linearly inseparable computation.

## Data availability

No data are associated with this article.

## Software availability

• Source code available from: https://github.com/rcaze/21_03Ca/tree/1.
• Archived source code: https://doi.org/10.5281/zenodo.6594665. ^11^
• License: MIT license.
