# Supplementary material for: All neurons can perform linearly non-separable computations
Source: F1000Res. 2022 Jun 8;10:539. Originally published 2021 Jul 6. [Version 3] doi: 10.12688/f1000research.53961.3 (PMC9198478; doi:10.12688/f1000research.53961.3)
Supplement: Peer review history

# Peer review: Reviewer response for version 3

**DOI:** 10.5256/f1000research.134167.r140000

**Published:** 2022-06-14

**Authors:** Balazs B Ujfalussy

**Affiliations:** 1 Laboratory of Biological Computation, Institute of Experimental Medicine, Budapest, Hungary

In general I accept the author's response to my comments. I have only 2 minor comments, that I list below.

- The authors renamed the model the Saturating Integrate and Fire, but the Caption of Fig 1 starts with "Dendrited IF". I think this should be corrected for making it consistent with the rest of the paper.
- In Eq. 2 g_D_1(t) has explicit temporal dependence, g_D_2 does not. Is there any reason for this or it is a simple typo? Also, I would consider denoting the two groups with roman numbers (I and II) of letters (a and b), since arabic numbers are for the inputs (1-4) which can be confusing.

Is the work clearly and accurately presented and does it cite the current literature?

Partly

If applicable, is the statistical analysis and its interpretation appropriate?

Not applicable

Are all the source data underlying the results available to ensure full reproducibility?

Yes

Is the study design appropriate and is the work technically sound?

Partly

Are the conclusions drawn adequately supported by the results?

Yes

Are sufficient details of methods and analysis provided to allow replication by others?

Yes

Reviewer Expertise:

Computational Neuroscience

I confirm that I have read this submission and believe that I have an appropriate level of expertise to confirm that it is of an acceptable scientific standard.

# Peer review: Reviewer response for version 2

**DOI:** 10.5256/f1000research.77421.r121426

**Published:** 2022-02-15

**Authors:** Balazs B Ujfalussy

**Affiliations:** 1 Laboratory of Biological Computation, Institute of Experimental Medicine, Budapest, Hungary

In this report the author extends his previous work (ref 3.) demonstrating that simple neurons with two saturating nonlinearities can implement certain non-trivial computational problems, i.e., the feature binding problem (FBP). The novelty of the current implementation is that it places the nonlinearity to the synaptic conductance term in the input instead of to the reduction of the synaptic driving force. This way the FBP can be implemented with electrically compact neurons without the need for independent electrical subunits.

**I have two main concerns:**

- Although the idea that nonlinear integration between different input streams could be implemented at the synaptic conductance level, actual experimental data supporting this hypothesis is not cited.
- The authors introduce the Dendritic Integrate and Fire model as a variant of the Leaky Integrate and Fire harboring multiple groups of interacting synaptic conductance. Since the model does not assume that the sites target physically separate dendritic compartments, I found the name potentially misleading.

**Specific comments:**

- The introduction states that cerebellar granule cells can be decomposed into multiple independent subunits, but the reference cited [ref. 8] does not directly imply this.
- In Eq. 1. I_s is input current and not input conductance
- I suggest to highlight that g in Eq. 2. denotes the total synaptic conductance associated with a group of input synapses, which is quite unusual assumption for most modelers.
- Fig. 1: in panel B grey and black denote the DIF and LIF models. In panel C the same colors denote clustered versus scattered configurations, according to the legend.
- Fig 1C: It is unclear what were the inputs used here: As far as I understand, there are 4 spike trains, 2 of which the input frequency F > 50 Hz and 2 with F < 50 Hz. What are the actual frequencies? Are these Poisson trains? Based on the response, I see a strong 60 Hz drive, but I don't see any other periodicity in the response. I would consider showing the timing of the inputs with green and blue ticks above the response.
- Fig. 1C: It is hard to see the spikes on the response - I would consider showing a larger y-axis range.
- Fig. 1C: It seems to me that the membrane potential is reset after each spike, but details of this reset are missing.
- Fig 1, legend: It is not explained how reference 5 is related to the figure.
- It is not clear why cFBP is compact. It is an n=4-dimensional problem, so it is defined by its 2^4=16 input-output pairs. Even if we restrict ourselves to the mappings with exactly 2 of the inputs being active, there are 6 of such pairs. I understand that it remains linearly non-separable no matter how we define the remaining two mappings, but the definition ('compact because they have four input/output lines') still feels somewhat vague and arbitrary.
- The statement 'a reduction in driving force does not generate sublinear summation in a DIF' is false. Reduction of driving force would generate sublinear summation even in a DIF. What the author might want to say is that in this particular example sublinear integration was not associated with reduction of driving force.
- Last paragraph: It is unclear how the proposed experiment would test whether the granule cells implementing cFBP use saturating input conductance or driving force reduction as a biophysical mechanism to solve the FBP.

Is the work clearly and accurately presented and does it cite the current literature?

Partly

If applicable, is the statistical analysis and its interpretation appropriate?

Not applicable

Are all the source data underlying the results available to ensure full reproducibility?

Yes

Is the study design appropriate and is the work technically sound?

Partly

Are the conclusions drawn adequately supported by the results?

Yes

Are sufficient details of methods and analysis provided to allow replication by others?

Yes

Reviewer Expertise:

Computational Neuroscience

I confirm that I have read this submission and believe that I have an appropriate level of expertise to confirm that it is of an acceptable scientific standard, however I have significant reservations, as outlined above.

# Response

**Published:** 2022-06-10

**Authors:** Romain Cazé

**Affiliations:** CNRS UMR 8520, France

*Although the idea that nonlinear integration between different input streams could be implemented at the synaptic conductance level, actual experimental data supporting this hypothesis is not cited.*

 - I proposed glutamate spillover as a candidate where nearby synapses interact. However, its effect on firing was never investigated to our knowledge. This work thus make a strong experimental prediction and give a computational role for glutamate spillover. I added two references to further support this argument.    

*The authors introduce the Dendritic Integrate and Fire model as a variant of the Leaky Integrate and Fire harboring multiple groups of interacting synaptic conductance. Since the model does not assume that the sites target physically separate dendritic compartments, I found the name potentially misleading.*

 - I renamed our model the Saturating Integrate and Fire, this model account for a neuron where synapses target distinct points where they interact. I now discuss this statement extensively in our conclusion.

*Specific comments:*

*The introduction states that cerebellar granule cells can be decomposed into multiple independent subunits, but the reference cited [ref. 8] does not directly* imply this.

 - I precised that ref.6 concerns pyramidal neurons only and not granule cells. I also insist in the introduction on the fact that granule cells are isopotential structure impossibl to decompose into subunits.

*In Eq. 1. I_s is input current and not input conductance*

 - I corrected the mistake

      

*I suggest to highlight that g in Eq. 2. denotes the total synaptic conductance associated with a group of input synapses, which is quite unusual assumption for most modelers.*

**

 - I now underline this crucial difference and discuss it in our conclusion

     Fig. 1: in panel B grey and black denote the DIF and LIF models. In panel C the same colors denote clustered versus scattered configurations, according to the legend.

      

 - I redraw the panel B and C of this figure to make it clearer.

*Fig 1C: It is unclear what were the inputs used here: As far as I understand, there are 4 spike trains, 2 of which the input frequency F > 50 Hz and 2 with F < 50 Hz. What are the actual frequencies? Are these Poisson trains? Based on the response, I see a strong 60 Hz drive, but I don't see any other periodicity in the response. I would consider showing the timing of the inputs with green and blue ticks above the response.*

 - I now explicit this point in the figure legend.

*Fig. 1C: It is hard to see the spikes on the response - I would consider showing a larger y-axis range.*

 - Spikes arised when we reached -62mV

      

*Fig. 1C: It seems to me that the membrane potential is reset after each spike, but details of this reset are missing.*

 - I precised the reset voltage in the method section.

      

*Fig 1, legend: It is not explained how reference 5 is related to the figure.*

 - This no longer apply in the current version of the manuscript

**

*It is not clear why cFBP is compact. It is an n=4-dimensional problem, so it is defined by its 2^4=16 input-output pairs. Even if we restrict ourselves to the mappings with exactly 2 of the inputs being active, there are 6 of such pairs. I understand that it remains linearly non-separable no matter how we define the remaining two mappings, but the definition ('compact because they have four input/output lines') still feels somewhat vague and arbitrary.*

 - The cFBP is compact because only four input lines need to be defined all the other remaining 12 can take any other value, we now explain this in the result section

      

*The statement 'a reduction in driving force does not generate sublinear summation in a DIF' is false. Reduction of driving force would generate sublinear summation even in a DIF. What the author might want to say is that in this particular example sublinear integration was not associated with reduction of driving force.*

 - The reduction in driving force would be insufficient in an isopotential neuron as it would affect the independence of g_1 and g_2. I now further underline this point in the discussion.

      

*Last paragraph: It is unclear how the proposed experiment would test whether the granule cells implementing cFBP use saturating input conductance or driving force reduction as a biophysical mechanism to solve the FBP.*

 -  I agree the experiment would only demonstrate that granule cells are capable of linearly inseparable computation. However, given their isopotential structure it is unlikely that saturation is due to a localized reduction in driving force. This point is now further emphasised in the discussion.

# Peer review: Reviewer response for version 2

**DOI:** 10.5256/f1000research.77421.r94490

**Published:** 2021-10-13

**Authors:** Athanasia Papoutsi, Spyridon Chavlis

**Affiliations:** 1 Institute of Molecular Biology & Biotechnology, Foundation for Research & Technology - Hellas, Heraklion, Greece; 2 Institute of Molecular Biology & Biotechnology, Foundation for Research & Technology, Hellas, Heraklion, Greece

We appreciate the clarifications/corrections made by the author and we support this work for indexing. We have some minor comments that do not change the impact of the work.  

- Page 5: “You can observe on Figure 1 that locally bounding g enables implement the of cFBP”. Consider removing the ‘of’ preposition.
- Figure Legend: “filled circles stand for a >50 Hz input spike train while empty circles stand for >50 Hz input spike train.”, please change the second ">"  to "<".
- Figure 1B and C: In Figure 1B, the color scheme denotes the LIF vs. DIF models. The same color scheme is used in panel C to denote the scattered vs. clustered case. As it stands now, is confusing. Consider changing the line style on panel C.
- Figure Legend: “In the clustered case (grey), the neuron reach spike threshold three times whereas it reaches spike thresold seven times in the scattered case (black).”  Please correct “thresold” to “threshold”. Also, from the figure, in the in the cluster case it reaches the spike threshold two times (not three).
- Please update the Github link to point to the revised code.
- There is a mismatch with code and text: code lines 71 and 77: the g is bounded between 0 and 0.1 nS (i.e., 100pS), while in the text referred to as 10pS.
- Figure 1 Legend: We suggest adding the respective episodes of the truth table in Table 1.
- Definition 2: the number of variables denoted with n should be in italic for consistency.

Is the work clearly and accurately presented and does it cite the current literature?

No

If applicable, is the statistical analysis and its interpretation appropriate?

Yes

Are all the source data underlying the results available to ensure full reproducibility?

Yes

Is the study design appropriate and is the work technically sound?

Yes

Are the conclusions drawn adequately supported by the results?

Yes

Are sufficient details of methods and analysis provided to allow replication by others?

Yes

Reviewer Expertise:

computational neuroscience; dendritic computations

We confirm that we have read this submission and believe that we have an appropriate level of expertise to confirm that it is of an acceptable scientific standard.

# Response

**Published:** 2022-02-01

**Authors:** Romain Cazé

**Affiliations:** CNRS UMR 8520, France

We will respond thoroughly to these minor comments as we receive new review reports. We would like to direct your attention to an important point. This work is based on a strong prediction/hypothesis: that multiple presynaptic neurons target the same postsynaptic receptor, which gives **a new role for glutamate spillover.**

# Response

**Published:** 2022-06-10

**Authors:** Romain Cazé

**Affiliations:** CNRS UMR 8520, France

*Page 5: “You can observe on Figure 1 that locally bounding g enables implement the of cFBP”. Consider removing the ‘of’ preposition.*

*Figure Legend: “filled circles stand for a >50 Hz input spike train while empty circles stand for >50 Hz input spike train.”, please change the second ">"  to "<".*

*Definition 2: the number of variables de*noted with n should be in italic for consistency.

 - I corrected these mistakes which were introduced during the editorial process

*Figure 1B and C: In Figure 1B, the color scheme denotes the LIF vs. DIF models. The same color scheme is used in panel C to denote the scattered vs. clustered case. As it stands now, is confusing. Consider changing the line style on panel C.*

      

 - I worked on th *e figure so panel B and C do not conflict we each other anymore*

*Figure Legend: “In the clustered case (grey*

*), the neuron reach spike threshold three times whereas it reaches spike thresold seven times in the scattered case (black).”  Please correct “thresold” to “threshold”. Also, from the figure, in the in the cluster case it reaches the spike threshold two times (not three).*

 - The panel has now changed and we rewrote the legend.

*Please update the Github link to point to the revised code.*

**

*There is a mismatch with code and text: code lines 71 and 77: the g is bounded between 0 and 0.1 nS (i.e., 100pS), while in the text referred to as 10pS*.

 - I updated the git repo and corrected the text to solve this mismatch

      

*Figure 1 Legend: We suggest adding the respective episodes of the truth table in Table 1.*

 - I followed the reviewer's suggestion.

    

# Peer review: Reviewer response for version 1

**DOI:** 10.5256/f1000research.57398.r89096

**Published:** 2021-07-13

**Authors:** Athanasia Papoutsi

**Affiliations:** 1 Institute of Molecular Biology & Biotechnology, Foundation for Research & Technology - Hellas, Heraklion, Greece

This Brief Report shows at a conceptual level that electrically compact neurons can solve non-linearly separable computations of four or more inputs. This is a result of the saturating responses to ‘clustered’ input at the dendritic level (simulating mainly the reduction of the driving force in the dendrites), and increased response to ‘scattered’ input at the somatic level. This study expands on previous work of the author and others and adds on the range of computations neurons can perform with their dendrites.

 We have two major concerns that limit the clarity of this work:

1. Figure 1B and 3rd paragraph on page 5: The x-axis label states ‘Time’, and the relevant text states that “the membrane voltage takes more time to reach threshold in the clustered case (total g = 10pS) than in the scattered case (total g = 20pS)”. Given that this work is based on an arbitrary thresholding of the output frequency, it is not obvious where time is involved and its meaning in the x-axis.
2. In the provided code on GitHub (line 78 in the code), the ceiling of the second dendrite (i.e., syn2 in the code) is set to 0.5 and not to 0.1. Please clarify the value used. If those different saturating thresholds were indeed used, this should be explicitly stated and reasoned in the main text.

Minor comments (not in order of importance nor appearance in the manuscript):

1. For clarity, specify that granule cells refer to the cerebellum (and not the hippocampus).
2. Correct R units to be MΩ (not mΩ).
3. Figure 1 legend: “filled circles stand for a >50 Hz input spike train while empty circles stand for >50 Hz input spike train.” Change the second > to <.
4. Page 4: “This computation that we call the the compact feature binding problem (cFBP) is linearly inseparable.” Delete the second ‘the’.

Is the work clearly and accurately presented and does it cite the current literature?

No

If applicable, is the statistical analysis and its interpretation appropriate?

Yes

Are all the source data underlying the results available to ensure full reproducibility?

Yes

Is the study design appropriate and is the work technically sound?

Yes

Are the conclusions drawn adequately supported by the results?

Yes

Are sufficient details of methods and analysis provided to allow replication by others?

Yes

Reviewer Expertise:

computational neuroscience; dendritic computations

I confirm that I have read this submission and believe that I have an appropriate level of expertise to confirm that it is of an acceptable scientific standard, however I have significant reservations, as outlined above.
